# Postictal suppression in electroconvulsive therapy (ECT) according to sex, age, diagnosis and treatment phase

**DOI:** 10.1192/j.eurpsy.2021.2070

**Published:** 2021-08-13

**Authors:** V. Llorca-Bofí, E. Buil-Reiné, M. Adrados-Pérez, G. Torterolo, M. Sánchez, A. Gisbert-Solà, S. Pàmpols-Pérez, R. Palacios-Garrán, A. Torrent, I. Batalla

**Affiliations:** Psychiatry Department, HU Santa Maria, Lleida, Spain

**Keywords:** Electroconvulsive therapy, postictal suppression

## Abstract

**Introduction:**

Postictal suppression (PSI) is considered a key feature for ECT’s outcomes because higher values have been correlated with clinical efficacy. However, little is known about the demographic factors influencing this parameter.

**Objectives:**

To analyze the influence of sex, age, diagnosis and treatment phase on ECT efficacy measured with PSI value.

**Methods:**

3251 ECT sessions were performed on 182 patients during two years at a university hospital. PSI was retrospectively analyzed comparing it according to sex (male, female), age, main diagnosis (major depressive disorder [MDD], bipolar disorder [BD], schizoaffective disorder [SZA], schizophrenia [SCZ]) and treatment phase (acute [a-ECT], continuation [c-ECT], maintenance [m-ECT]).

**Results:**

PSI values were 69.76 % (SD 17.05) in women and 70.72 % (SD 16.81) in men without differences between sexes (F=0.979; p=0.607). PSI was correlated with age (r=-0.058; p=0.031). MDD PSI was 70.01 % (SD 16.88), for BD it was 69.48 % (SD 17.00), for SZA it was 68.62 % (SD 17.39), and for SCZ it was 70.73 % (SD 17.18), without differences between diagnosis (F=1.085; p=0.141). According to treatment phase, PSI in the a-ECT was 72.26 % (SD 16.43), in the c-ECT it was 67.83 % (SD 17.53), and in the m-ECT it was 68.47 % (SD 17.02), without differences between phases (F=0.901; p=0.915).

**Conclusions:**

Although there exist statistically significant association between age and PSI it is a negligible correlation with no clinical relevance. Thus, we conclude that neither sex nor age, nor diagnosis, nor treatment phase seem to influence PSI to a relevant degree.

**Disclosure:**

No significant relationships.

